# Characterization of Histone H3 Gene Family Reveals That *GmHH3-3* is Associated With Higher Seed Weight in *Glycine max*

**DOI:** 10.3389/fgene.2022.949027

**Published:** 2022-07-22

**Authors:** Chahat Fatima, Muhammad Hammad Nadeem Tahir, Rao Muhammad Ikram, Zulqurnain Khan, Muhammad Sajjad, Ghulam Qanmber, Essam Darwish, Zhide Geng, Gao Xiangkuo, Shoaib Ur Rehman

**Affiliations:** ^1^ Institute of Plant Breeding and Biotechnology, MNS University of Agriculture, Multan, Pakistan; ^2^ Department Agronomy, MNS University of Agriculture, Multan, Pakistan; ^3^ Department of Biosciences, COMSATS University Islamabad (CUI), Islamabad, Pakistan; ^4^ State Key Laboratory of Cotton Biology, Cotton Research Institute of Chinese Academy of Agricultural Sciences, Anyang, China; ^5^ Plant Physiology Section, Agricultural Botany Department, Faculty of Agriculture, Cairo University, Giza, Egypt; ^6^ Institute of Food Crops, Yunnan Academy of Agricultural Sciences, Kunming, China

**Keywords:** histone H3, marker-assisted breeding, KASP, drought, *Glycine max*

## Abstract

The main function of histone protein is to provide support to the structure of chromosomes. It helps in binding a long thread of DNA into a more condensed shape to fit into the nucleus. From histone variants, histone H3 (*HH3*) plays a crucial role in plant growth and development. Characterization of histones has not been reported in *Glycine max* till now. The objective of this study was to characterize the *HH3* gene family for molecular breeding of *G. max*. In this study, 17 *HH3* members in *G. max* were identified by performing local BLASTp using *HH3* members from *Arabidopsis* as a query. Phylogenetic analysis classified *HH3* genes in seven clades. Sequence logo analysis among *Arabidopsis thaliana*, *Oryza sativa*, and *Glycine max* showed a higher level of similarity in amino acids. Furthermore, conserveness of *G. max HH3* genes was also confirmed by Gene Structure Display. Ten paralogous gene pairs were identified in *GmHH3* genes in the *Glycine max* genome by conducting collinearity analysis. *G. max HH3* genes have experienced strong purifying selection pressure, with limited functional divergence originating from the segmental and whole-genome duplication, as evidenced by the *Ka/Ks* ratio. The KASP marker was developed for *GmHH3-3* gene. Genotyping was performed on 46 *G. max* genotypes. This differentiation was based upon the presence of either *GmHH3-3-C* or *GmHH3-3-T* allele in the CDS region. The results showed that *G. max* accessions containing the *GmHH3-3-T* allele at respective locus showed higher thousand seed weight than that of those accessions that contain the *GmHH3-3-C* allele. This research provides the basic information to further decipher the function of *HH3* in soybean.

## Introduction

Histone proteins provide structural support to chromosomes and assist in binding long strands of DNA into a more compressed shape to reside in the nucleus. Some of the histone variants are associated with gene expression regulation. These DNA folding proteins are present in the chromosomes of all studied eukaryotic cells. Histones are highly conserved and are categorized into five extensive classes named *HH1/H5*, *HH2A*, *HH2B, HH3*, and *HH4*. On the basis of gene expression analysis, histone genes are classified into three basic classes, replication-dependent histones, replication-independent histones, and tissue-specific histones (Elsaesser, Goldberg, and Allis, 2010). The replication-dependent histones express highly just before the initiation of S-phase and are suppressed at the termination of DNA replication, while during the whole cell cycle, the replication-independent histone variants continuously express themselves; hence, they are also named as replacement histones (Filipescu, Müller, and Almouzni, 2014).

Mainly, all histone proteins are involved in the folding of chromosomes, but *HH3* is associated with the chromosome structure (Bhasin, Reinherz, and Reche, 2006). In the case of histone H3, three different strains of *HH3* proteins are found in both animals and plants: H3.1, H3.3, and the centromere-specific CENP-A (CENH3) (Stroud et al., 2012). H3.1 and H3.3 have the same length and amino acid sequence, while the CenH3 variants differ from these two by a large and extensive tail at the N-terminal (Malik and Henikoff, 2003).

The role of H3 protein in transcription has been demonstrated by genome-wide profiling of histone H3.3 variants in mammalian *Drosophila* (Deal, Henikoff, and Henikoff, 2010; Goldberg et al., 2011). Enrichment of H3.3 in pericentric heterochromatin and telomers has also been explored by other research (Wong et al., 2010). Histone H3 protein plays its peculiar role in distinct functions involving gene silencing, gene inactivation, genomic instability, and sex chromosome inactivation (Celeste et al., 2003; Fernandez-Capetillo et al., 2003). Genome-wide identification in *Arabidopsis* resulted in the identification of a male gamete–specific gene named *AtMGH3* (Okada et al., 2005). Similarly, genome-wide analysis of two *HH3* variants *HH3.1* and *HH3.3* highlighted similar genomic localization schemes with certain unique attributes in *Arabidopsis* (Stroud et al., 2012). H3.3 has been shown to be highly correlated with transcriptional activity in the transcribed regions, although H3.3 at promoters is often unrelated to transcription. (Shu et al., 2014). Genome-wide characterization of HH3 in cotton reported that *GhHH3* genes were most appropriately expressed in the tissues of the ovule (Qanmber et al., 2019a). At present, no comprehensive report on the characterization of the *HH3* gene family in soybean is available. We believe that this gene family has the potential to be used in the molecular breeding of soybean.

Soybean (*G. max*) is a leguminous crop with prime economic importance. Several studies reported the phenotypic differences between *G. max* and *G. soja* (ancestor of *G. max*), but both the species have the same number of chromosomes with normal meiotic chromosomal pairing and are cross-compatible.

In accordance with the importance of soybean, the present study aims for genome-wide characterization of the histone H3 gene family in *G. max*. This gene family has been broadly characterized in *Gossypium hirsutum* (Du et al., 2006; Qanmber et al., 2019a), *Arabidopsis thaliana* (Okada et al., 2005; Stroud et al., 2012), and *Oryza sativa* (Du et al., 2006; Hu and Lai, 2015), but currently there is no extensive and detailed study reporting the genome-wide characterization of *HH3* genes in *G. max*. The whole-genome sequencing of soybean in the past decade (Wollmann et al., 2012; Qi et al., 2014) has opened the way to study various gene families in soybean by using modern genome-wide approaches. The availability of pan-genome is expected to pave the way for molecular breeding in soybean. Although quantitative trait loci and SNP-based markers have been reported (Seo et al., 2022), continuous searching for genes underpinning yield and its director contributing traits should continue for sustainable development in the research sector.

In the current study, we identified *HH3* gene members in *G. max*. Gene structure, gene duplication *via* collinearity analysis, sequence logo analysis, chromosome duplication, and domain architecture were predicted by using different structural and functional approaches. Evolutionary analysis was also performed by constructing a phylogenetic tree. Tissue-specific expression analysis was also checked, and a heat map was constructed based on the fragments per kilobase of transcript per million mapped reads (FPKM) values. An SNP-based high-throughput KASP molecular marker for the candidate gene *GmHH3-3* was also developed by exploring the pan-genome of soybean.

## Materials and Methods

### Sequence Identification

*AtHH3* protein sequences were used as a query to retrieve the sequences of HH3 from *G. max. HH3* protein sequences were also extracted from other species by using the respective databases for *Gossypium raimondii* (V-2.0), *Solanum tuberosum* (V-10), *Theobroma cacao* (V-10), *Oryza sativa* (V-10), *Zea mays* (V-10), *Chlamydomonas reinhardtii* (V-5.5), *Selaginella moellendorffii* (V-1.0), *Ananas comosus* (V-3.0) *Vitis vinifera* (V-10), *Chlamydomonas reinhardtii* (V-5.5), and *Cicer arietinum* (V-2.0). Local BLASTp search was performed to extract the desired sequences. Databases for all organisms were extracted from Phytozome v11 (https://phytozome.jgi. doe.gov/pz/portal.html). For further confirmation of the retrieved *HH3* protein sequences, bioinformatics techniques including InterProScan 63.0 (Jones et al., 2014) (http://www.ebi.ac.uk/InterProScan/) and SMART (Letunic, Doerks, and Bork, 2015) (http://smart.embl-heidelberg.de/) were used. Biophysical properties such as isoelectric point, protein length, and molecular weight were computed by using the ExPASy ProtParam tool (https://web.expasy.org/protparam/). Sub-cellular localization was predicted by using Softberry (http://www.softberry.com/).

### Conserved Sequence and Phylogenetic Analysis

In order to perform phylogenetic analyses, complete protein sequences of *HH3* genes of the aforementioned species were extracted from the Phytozome (https://phytozome.jgi. doe. gov/pz/portal.html). For the construction of a phylogenetic tree, ClustalW program from MEGA-X (Kumar et al., 2018) was used to perform sequence alignment, and then the tree was generated using the maximum likelihood method. Amino acid sequences of *A. thaliana*, *O. sativa*, and *G. max* were aligned by multiple sequence alignment using Clustal X 2.0 (http://www.clustal.org/clustal2/) to create a sequence. Logos were generated by using the online tool WEBLOG (Crooks et al., 2004).

### Domain Architecture, Gene Structure, and *Cis*-element Analysis

To perform domain architecture analyses, the full-length protein sequences of *GmHH3* genes were subjected to MEME software (Crooks et al., 2004) (https://meme-suite.org/meme/tools/meme), as described in previous studies (Li et al., 2019). For gene structure analyses, genomic and conserved DNA sequences were downloaded from the Phytozome and Newick file obtained by aligning protein sequences in MEGA-X using the CLUSTAL-W approach. This Newick file genomic and CDS sequences were subjected to GSDS 2.0 (Hu et al., 2015). The PlantCARE database (Li et al., 2019) was used to analyze *cis*-elements up to the 2 kb promoter region, and anticipated *cis*-elements were categorized in accordance with their functional divergence, as stated previously (Pandey et al., 2016).

### Chromosomal Localizations, *Ka/Ks* Ratio, and Collinearity Analysis

Chromosomal mapping of *GmHH3* genes was identified first by the soybean genome annotation file (https://www.soybase.org/genomeannotation/), and then we extracted gff3-files. Paralogous gene pair data were obtained from collinearity analysis as described earlier (Yang et al., 2017), and then a figure was created by using CIRCOS (Krzywinski et al., 2009) to express the outcomes of synteny analysis. Duplicated gene pair sequences were aligned by using Clustal X 2.0, and synonymous and non-synonymous (*Ks*, *Ka*) and divergence level ratios were measured. Finally, *Ka* and *Ks* values were computed using the CODEML program by using the PAML package (Yang, 2007) and used to determine dispersed, segmental, and/or whole-genome duplication in soybean for *GmHH3*.

### Tissue Specific Expression Pattern of *GmHH3*

To investigate the gene expression pattern of *GmHH3* in different tissues at different growth stages, the FPKM values were extracted from the ePlant/soybase database (https://bar.utoronto.ca/eplant_soybean/). After taking log10 of each FPKM value, a heat map was constructed using TB-Tools (Chen et al., 2003) to express the transcript level of *GmHH3* genes based on (FPKM) values.

### Isolation of Candidate *GmHH3* Genes From Soybean PAN-Genome

The PAN-genome was used to identify polymorphic sites in *GmHH3s*. For this, the whole-genome sequences (WGSs) of three cultivars of *Glycine max* (Willliams-82 (Wm82. a4), Lee (Lee.a1), and Zhonghuang-13 (ZH13. a1) were downloaded from SoyBase (https://soybase.org/). Local BLAST was used to identify *GmHH3* sequences in the abovementioned three cultivars. The SeqMan program (Swindell, 1997) in the DNAstar Lasergene software package (Burland, 2000) was used for assembling of the genes to obtain the consensus sequence of every gene.

### Phenotyping and Genotyping

A set of 46 *G. max* accessions was collected from the gene bank of the MNS University of Agriculture, Multan, (MNSUAM). These accessions were planted in two different environments, that is, under “well water” and “water limited” conditions following an augmented design (check = UAM-SB-200) at the research farm of MNSUAM, Spring 2021. The “well water” experimental units were irrigated after an interval of ∼15 days, whereas for “water limited” experimental units, the soybean accessions were subjected to drought stress conditions, especially at the flowering stage. Each accession was planted on two beds on both sides. The dimensions of each bed were length × width = 15 × 2.5 ft. Seeds were planted with plant-to-plant distance of 1 ft with two seeds at one place, thinning was practiced to eradicate the unhealthy one, and healthy plants were retained. Phenotypic data were collected from six plants of each soybean accession for plant height (inches), number of pods plant^−1^, pod length (cm), number of seeds pod^−1^, seed weight plant^−1^ (g), thousand seed weight (g), seed length (mm), seed width (mm), and seed thickness (mm) from both water regime conditions.

The genomic DNA of the studied soybean germplasm was extracted from young leaves (one leaf per soybean accession) following the CTAB method (Aboul-Maaty and Oraby, 2019). DNA quality was initially checked by using a NANO-Drop (K5800C Micro-Spectrophotometer) followed by running the extracted DNA on 1% agarose gel. Out of 17 *GmHH3s*, only one gene (*GmHH3-3*) showed polymorphic site. CDS sequences of *GmHH3-3* along with SNP sites from three cultivars are given in (Supplementary Table 1). A typical KASP assay (http://www.lgcgenomics.com) was designed on the SNP present in the C-terminal region of the gene. From the PAN-genome, we came to know that Williams-82 possessed the *GmHH3-3-*T allele at 165 nt, while LEE and Zhonghuang-13 contained the *GmHH3-3-C* allele at 165 nt. Hence, a KASP assay on the SNP (at 165 nt *C/T*) was developed. Two allele-specific reverse primers and one common forward primer were designed to perform allele calling (Supplementary Table 1).

One reverse complement allele-specific primer was designed for “*T*” base (detected by FAM), and another reverse complement allele-specific primer was designed for “*C*” base (detected by HEX). DNA of some accessions (Williams-82 and Lee), in which the target gene had been sequenced, were initially selected to counter-check the reliability of molecular markers. The primer mixture contained 12 µl of each tailed primer (100 µM), 30 µl common primer (100 µM), and 46 µl double distilled water. A KASP assay was performed in 96-well PCR plates and set up ∼5 µl reaction mixture. The recipe of the mixture for 1X is given in (Supplementary Table 2). PCR conditions were as follows: hot start at 95°C for 15 minutes, followed by 10 touch-down cycles (95°C for 20 s; touch-down at 61°C initially and decreasing by 0.6°C cycle-1 for 25 s), followed by 32 more cycles of annealing (95°C for 15 s, 57°C for 1 min). Genotyping (PCR) was performed by using the CFX Connect Real-Time PCR detection system (Bio-Rad^®^ laboratories Inc. United States). The PCR plate was also read by the QuantStudio 7 Flex Real-Time PCR system.

### Association Analyses

Microsoft Excel 2019 was used to perform descriptive statistics and variance estimations. *P* 0.05 was used to determine whether a marker-trait correlation was statistically significant. The effects of each allele of *GmHH3-3* at 165 nt from CDS were also analyzed by using Student’s *t* test at *p* < 0.05.

## Results

### Sequence Identification

A total of 139 *HH3* proteins among 12 species were identified. Out of the studied 139 *HH3* members, 14 are from *Arabidopsis* (6-H3.1, 7-H3.3, and 1-centromeric variant); 13 members from *G. raimondii* (8-H3.1 and 5-H3.3 variants); 17 *G. max* members; 13 *O. sativa* members; nine *T. cacao* members; 14 *S. tuberosum* members*,* 12 *C. arietinum* members, 16 *Z. mays* members; 10 *A. comosus* members, 11 *C. reinhardtii* members, six *S. melanodorffii* members, and four *V. vinifera* members. We found that almost all selected plants have a minimum of 4-*HH3* genes, and from these *G. max* has the most 17) *HH3* genes, while *V. vinifera* has only four, demonstrating that *HH3* genes have undergone a bigger-scale expansion. (Supplementary Table 3). As our main focus was on *G. max,* so other biophysical properties were also determined including locus ID, coding sequence (CDS), gene length, molecular weight (MW), protein length, isoelectric point (pI), subcellular localization, and chromosome position (Supplementary Table 4). *GmHH3-7* had the maximum length of coding region (798 bp) followed by *GmHH3-15* which had 561 bp of coding sequence, while all other *GmHH3* members had coding sequence lengths of 411bp. However, it was predicted that all the *GmHH3* genes are localized in the nucleus.

### Sequence Alignment and Phylogenetic Analysis

**FIGURE 1:**
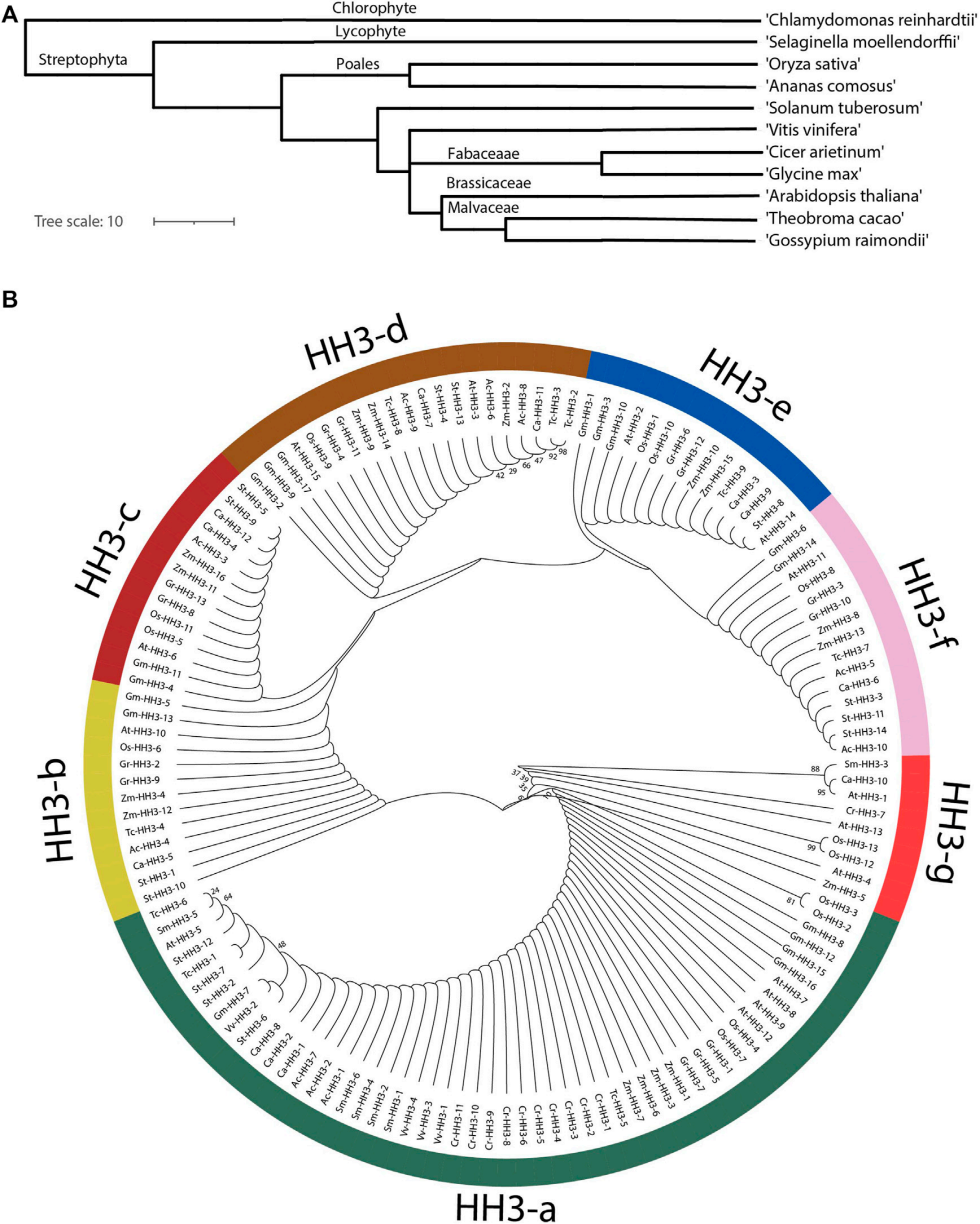
Phylogenetic analyses of the HH3 gene in different organisms. **(A)** Phylostratum analysis of HH3 genes. **(B)** Phylogenetic tree divided members of HH3 genes in seven clades; green color shows clade-a, yellow colors shows clade-b, maroon color shows clade-c, brown color shows clade-d, and blue color shows clade-e.

Phylostatum analysis was performed, and it showed that *HH3* genes were present in primitive plant ancestry as these genes are present in *C. reinhardti*, an older plant; lineage. *HH3* genes are located in monocots, dicots, lycophytes, chlorophytes, and angiosperms showing the large-scale expansion of *HH3* genes across the plant kingdom (Figure 1A). An evolutionary tree was constructed to estimate the deeper relation of *HH3* genes among the studied organisms including dicotyledons (*A. thaliana*, *G. raimondii*, *G. max*, *S. tuberosum*, *V. vinifera T. cacao*, and *C. arietinum*), monocotyledons (*O. sativa* and*, Z. mays*), *C. reinhardtii* (chlorophyte), *S. moellendorffii* (lycophyte), and *A. comosus* (angiosperm). The prefixes At, Gr, Gm, St, Tc, Vv, Ca, Zm, Cr, Sm, Os, and Ac were used in place of the names of *HH3* genes from *G. max*, *A. thaliana*, *G. raimondii*, *S. tuberosum*, *T, cacao*, *V. vinifera*, *C. arietinum*, *O. sativa*, *Z. mays*, *C. reinhardtii*, *S. melaonodorfii*, and *A. comosus,* respectively*.*

The evolutionary tree (Figure 1B) shows that all 139 genes from the studied organisms were naturally classified into seven Clades, that is, Clade a-g. Clade-a contains most of the *HH3* genes (53 genes) followed by clade-d (21 genes), clade-e and clade-f (15 genes), clade-c (14 genes), clade-b (13 genes), and clade-g (9 genes). *HH3* clade-a contains genes from all the studied species with maximum members showing that the *HH3* gene family is highly conserved among all species. Clade-b, d, and f contain members from *O. sativa*, *G. max*, *A. thaliana*, *G. raimondii*, *Z. mays*, *T. cacao*, *A. comosus*, *C, arietinum*, and *S. tuberosum*. Clade-c contains fourteen members from eight species excluding members from *T. cacao*, *C. reinhardtii*, *V. vinifera*, and *S. moellendorffii*. Clade-e contains 15 members from *S. tuberosum, G. raimondii, Z. mays, A. thaliana, T. cacao, O. sativa, and G. max;* Clade-g contains nine members from *A. thaliana, Z. mays, C. reinhardtii, S. moellendorffii, O. sativa,* and *C. arietinum.* All members from clade-g have evolved separately. All clades from a–g contain members from both monocots and dicots showing that the *HH3* gene family evolved before monocot and dicot separation. Phylogenetic analysis in this study showed gene enlargement in *G. max*. Moreover, orthologous gene pairs extracted from similar branch nodes were noticed in almost all studied species. During the evolutionary process, GmHH3 genes have undergone duplication events, which resulted in the paralogous gene pair’s formation, although this duplication was irregular in all clades and different studied organisms.

### Conserved Amino Acid Residue Analysis

**FIGURE 2:**
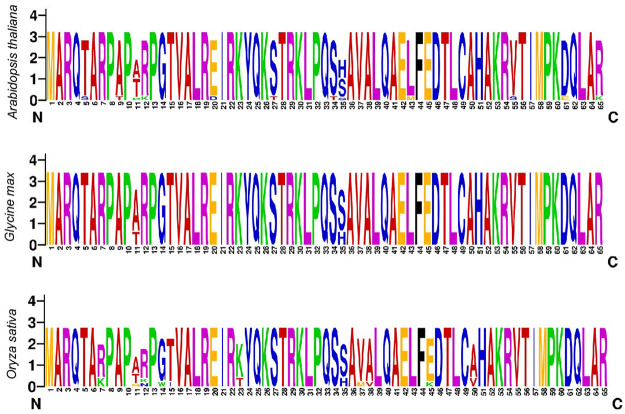
Sequence logos of conserved amino acid residues generated for three species.

To explore the amino acid residues (AARs), conservation multiple sequence alignment was conducted to perceive the homologous domain sequence in *GmHH3* genes. This alignment was conducted in model plants including *A. thaliana*, *O. sativa*, and in our studied crop *G. max*. Results showed high similarity ratio in logos of all three species. For example, few AARs including M [1], A [2], R [3], Q [4], A [6], R [7], P [8], P [10], P [13], G [14], T [15], V [16], A [17], L [18], R [19], I [21], R [22], K [23], Y [24], Q [25], K [26], T [28], R [29], K [30], L [31], P [32], Q [33], A [36], V [37], A [38], L [39], Q [40], A [41], E [42], F [44], E [45], D [46], T [47], L [48], C [49], A [50], H [51], A [52], K [53], 4 [54], T [56], I [57], M [58], P [59], K [60], Q [62], L [63], and A [64] were found to be highly conserved, showing that HH3 protein is having a highly conserved pattern of distribution without any discrimination of the N or C terminal (Figure 2).

### Chromosomal Localizations, *Ka/Ks* Ratio, and Collinearity Analysis

**FIGURE 3:**
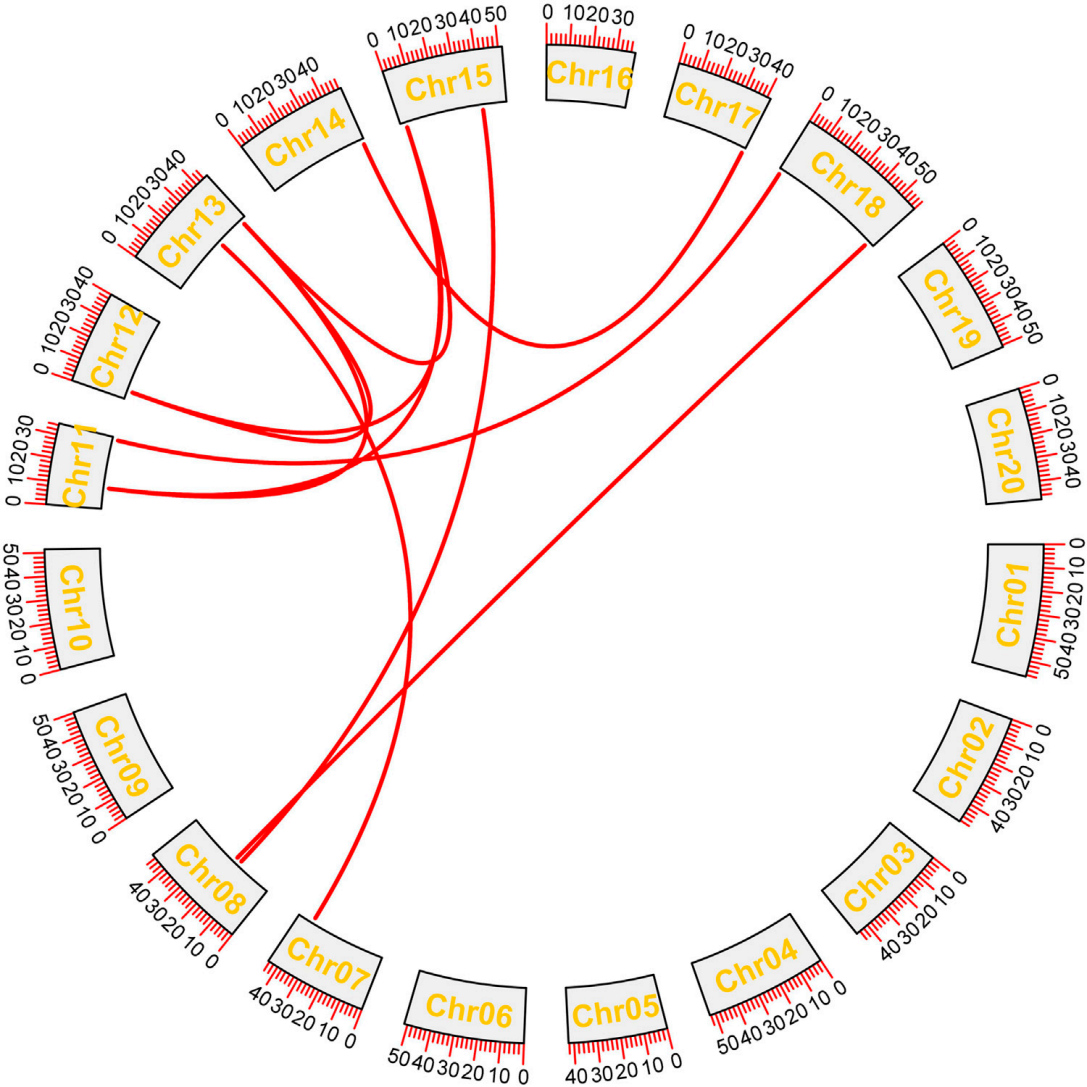
Gene duplication analyses of *GmHH3*. Red lines show the duplication of genes on different chromosomes.

The GFF3 files were utilized to map studied *GmHH3* genes onto their corresponding chromosomes. Paralogous gene pairs were identified in *G. max* in order to inspect the locus relationships among *GmHH3* genes, (Figure 3). The results of synteny analysis confirmed that most of the gene loci are significantly conserved. A total of 10 paralogous gene pairs were recognized, of which all studied *GmHH3* genes except *GmHH3-1*, *GmHH3-2*, *GmHH3-8*, and *GmHH3-16* had undergone whole-genome or segmental duplication (WGD). *GmHH3-1* and *GmHH3-2* showed dispersed duplication, while *GmHH3-8* and *GmHH3-16* showed tandem duplication (Supplementary Table 5). Non-functionalization, neo-functionalization, and sub-functionalization are the functional divergences in genes that can result during the process of evolution. The extent and nature of selection can be determined by calculating *Ka/Ks* values of these duplicated genes. In the case of neutral selection, the *Ka/Ks* value is always equal to 1; for positive selection, the *Ka/Ks* value is always greater than 1, while duplicated genes having *Ka/Ks* ratio less than 1 express the ability for purifying selection.

The results of our study showed that all *GmHH3* genes showed *Ka/Ks* values less than 1. We can conclude that *GmHH3* genes have experienced strong purifying selection pressure with a little functional deviation because of segmental and whole-genome duplication.

### Domain Architecture, Gene Structure, and *Cis*-element Analysis

The plantcare database was used to explore cis-elements present in 2 kb upstream of *GmHH3* genes. Results showed that all *GmHH3* genes carry various motifs for growth and development, light responses, and for several stress responses. For growth and development, important motifs are GCN-4 motif, TATA box, CAAT box, and circadian. GA-motif, GATA-motif, and Box-4 are for light responses. ABRE, AuxRR, MYB, and WUN–motif are for several stress responses. Various *cis-*elements performing their functions in different responses are given (Supplementary Table 6).

**FIGURE 4:**
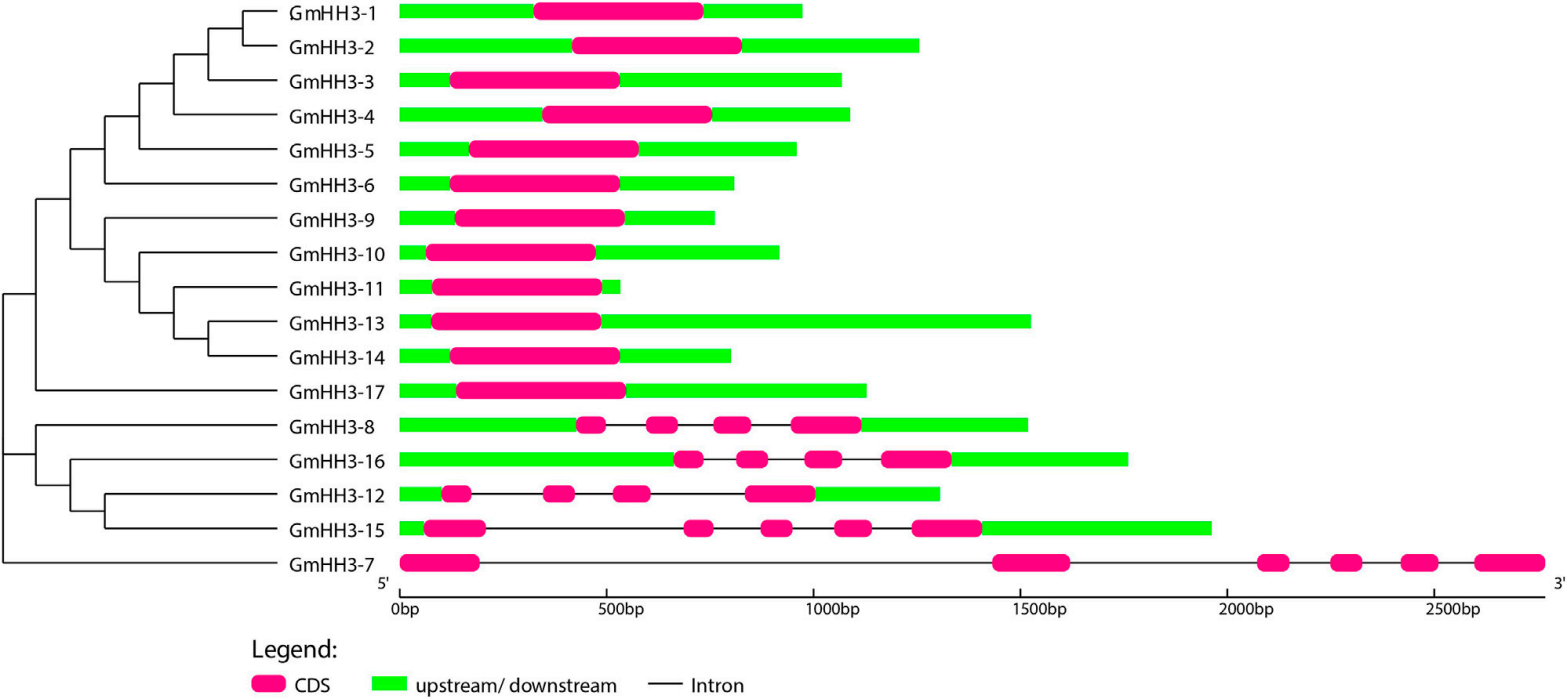
Exon/intron structure display of *GmHH3*. Pink box shows CDS regions, green box shows upstream/downstream regions, and black line shows introns.

**FIGURE 5:**
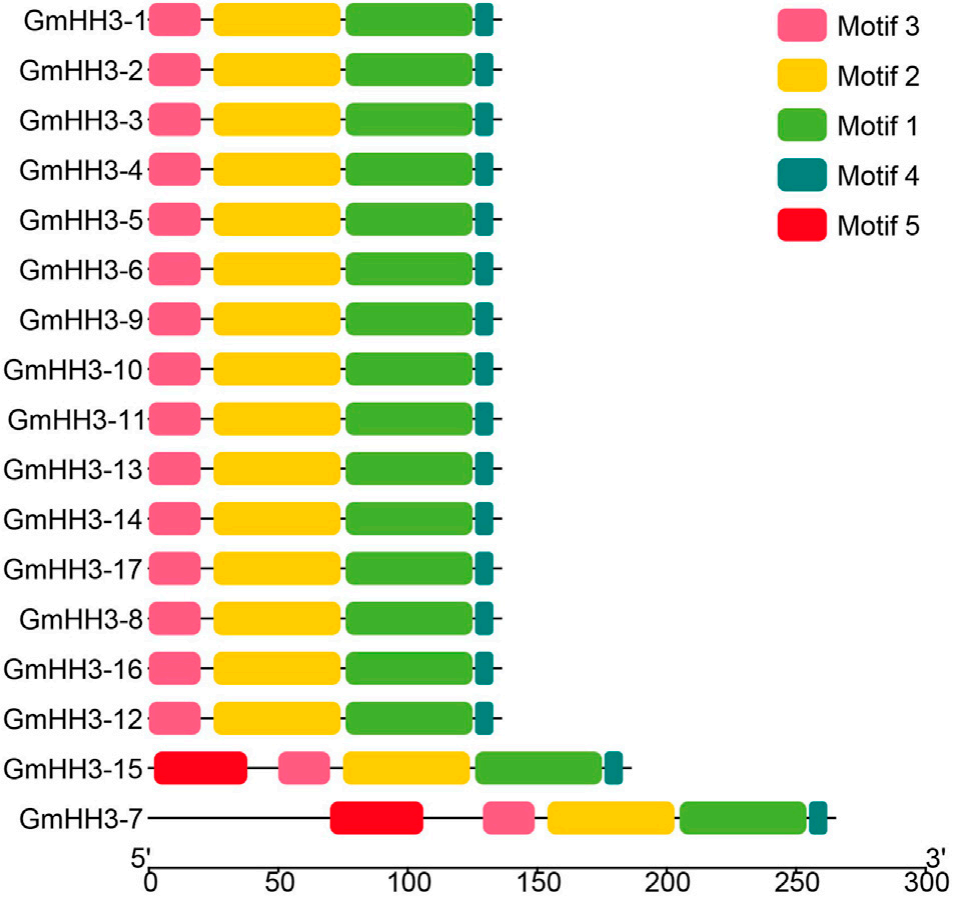
Conserved motifs in *GmHH3*.

The evolution of a plant species is always associated with its gene structure. In order to explore the evolutionary relationship of all *GmHH3*, the gene structure along with its phylogenetic tree was constructed (Figure 4). Out of 17 *GmHH3* members, 12 members have no introns and have only one exon. The remaining genes showed different exon/intron patterns (Figure 4). Moreover, all *GmHH3* genes showed a significantly similar motif pattern as all the genes except *GmHH3-15* and *GmHH3-7* have the same motifs. Overall, all *GmHH3* genes displayed a highly conserved pattern of motif distribution and gene structure (Figure 5).

### Expression Profiling of *GmHH3* Genes in Different Tissues

**FIGURE 6:**
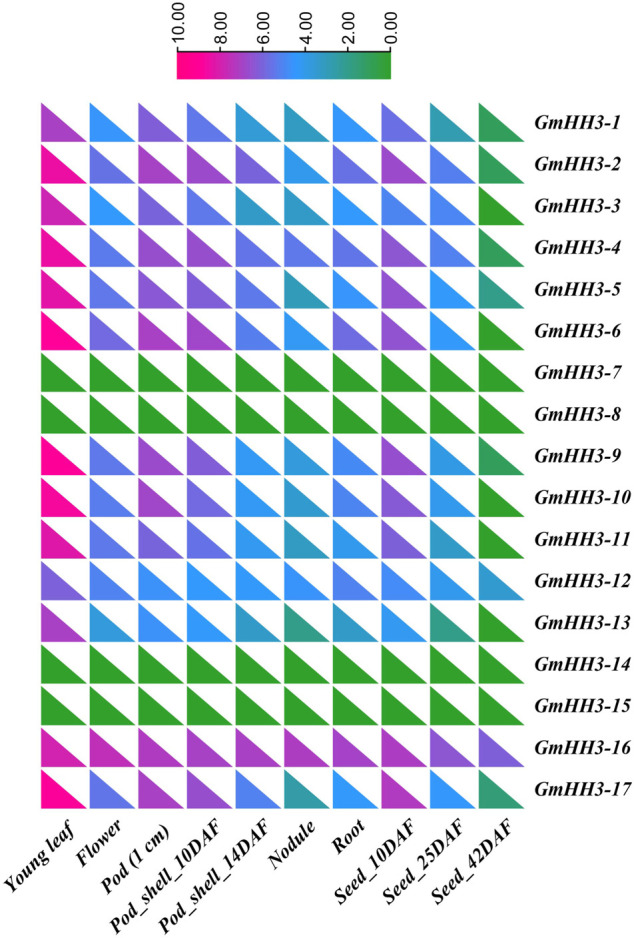
Tissue-specific expression profiling based on FPKM values. DAF, days after fertilization.

The biological function of a gene can be predicted by its expression. So, the expression of *GmHH3* genes was inspected in different soybean tissues based on FPKM values. To examine the expression of *GmHH3* genes in enormous plant tissues at different growth stages, the transcript level values were obtained from the ePlant/soybase database (https://bar.utoronto.ca/eplant_soybean/), and a heat map was created for different tissues in all 17 *GmHH3* genes (Figure 6). We observed that all *GmHH3* genes (except *GmHH3-7*, *GmHH3-8*, *GmHH3-14*, and *GmHH3-15*) were widely expressed in young leaves, and all genes except these four genes show their expression in all tissues. Data were recorded on the following parameters including young leaf, flower, pod (1 cm), pod shell 10 DAF (Days after flowering), nodule, root, pod shell (14 DAF), seed (10 DAF), and seed (25 DAF) explaining that *GmHH3* genes are involved in enormous biological functions. From all *GmHH3* genes, *GmHH3-*16 is showing higher expression than others*.* All of the genes with identical expression patterns were discovered to be clustered together.

### Sequence Polymorphism Assay and Development of the KASP Marker for *GmHH3-3*

**FIGURE 7:**
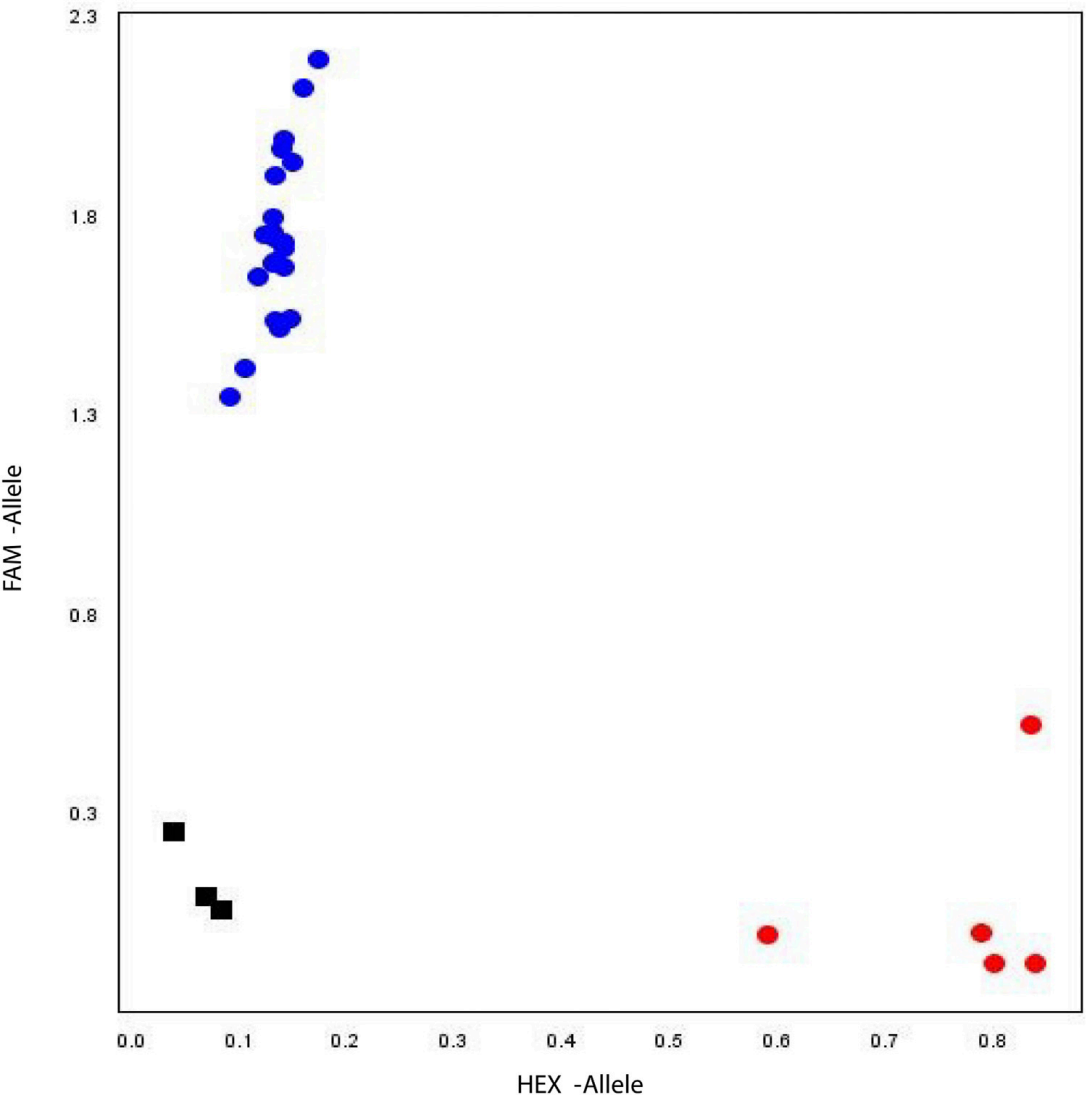
Allelic discrimination plot. KASP assay of *GmHH3-3* along *X*- and *Y*-axes. Blue dots represent accessions carrying the FAM type allele, and red dots show accessions having the HEX-type allele. Black box shows the non-template control.

The soybean PAN-genome was used to explore the polymorphic sites for all *GmHH3* genes. Except for *GmHH3-3*, no gene showed sequence polymorphism and hence were excluded from further marker-trait association analyses. For *GmHH3-3*, the SNP at 165 nt (*T/C*) was identified in exon with no change in amino acid. The scatter plot for the developed KASP assay displays the clustering of soybean accessions on *X-HEX* and *Y-FAM* axes. Accessions colored blue contain the *GmHH3-3-T* allele, whereas accessions colored red have the *GmHH3-3-C* allele (Figure 7).

### Association Analysis of *GmHH3-3* Allelic Variations and Morphological Traits

**FIGURE 8:**
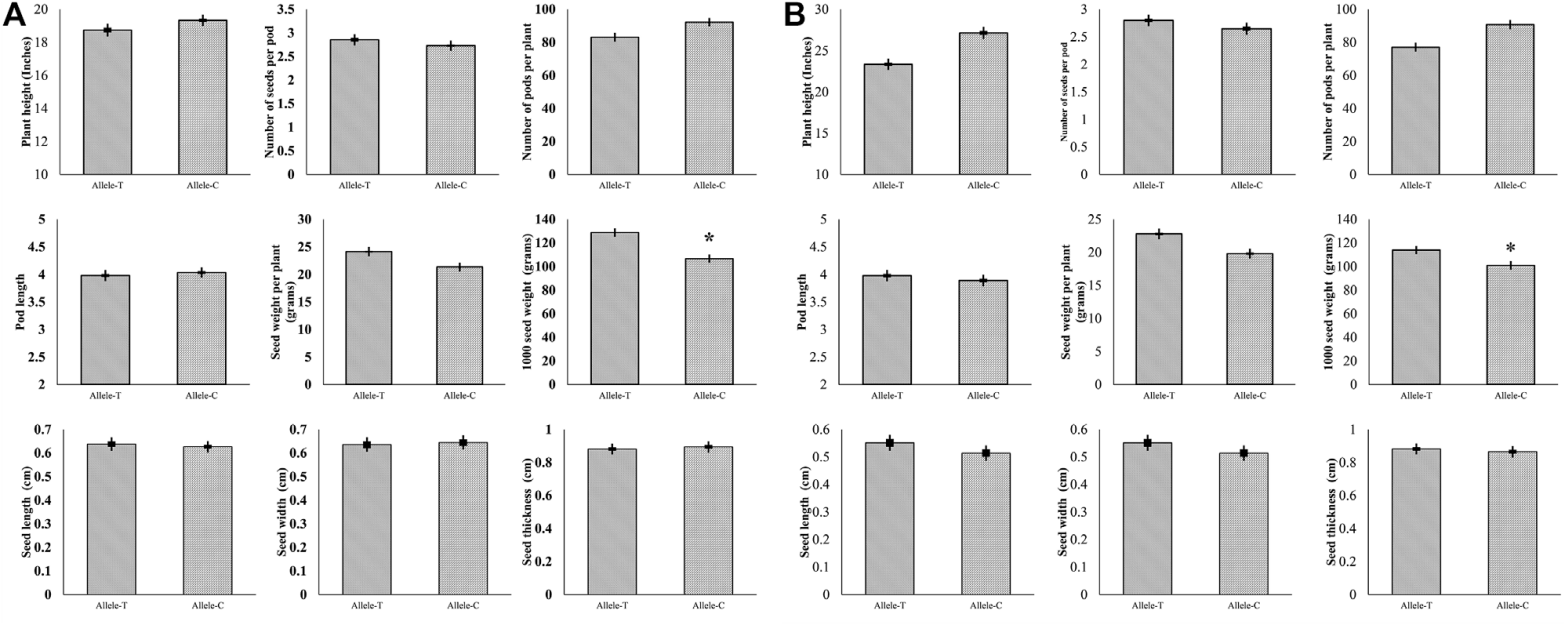
Phenotypic comparison of two GmHH3-3 allelic variations in two environments. **(A)** Phenotypic comparison under well-watered and **(B)** under water-limited conditions.

For *GmHH3-3*, 41.3% of the studied germplasm possessed *GmHH3-3-T*, while 58.6% possessed *GmHH3-3-C*. Association analysis was performed on all the aforementioned traits in both growing conditions, and non-significant statistical differences were recorded in all studied traits except for thousand seed weight. Association analysis exhibited that at unique field sites, the allele *T* of *GmHH3-3* was linked with higher thousand seed weight under both water regime conditions (Figure 8 A, B) indicating that the *GmHH3-3*-*T* allele has a superiority over *GmHH3-3-C* and hence can be used for future soybean breeding programs.

## Discussion

The biological function of *HH3* genes and histone modifications in various species has already been investigated in a number of publications. (Bhasin, Reinherz and Reche, 2006; Elsaesser, Goldberg and Allis, 2010; Wollmann et al., 2012; Hu and Lai, 2015). Till now, no comprehensive assessment of *G. max HH3* genes has been carried out. We presented a detailed analysis of *HH3* genes in *G. max* in order to investigate the role of the *HH3* gene *in G. max* and establish a platform for future research.

In our study, we performed evolutionary analysis of 12 different organisms including monocotyledons dicotyledons, chlorophytes, lycophytes, and angiosperms. The *HH3* gene family’s phylostratum analysis revealed the earliest plant lineage, with *HH3* genes found in *C. reinhardtii* (chlorophyte), showing that *HH3* genes came from the phylostratum of early land plants and that probable orthologous genes of *HH3* are found across the plant kingdom. All *HH3* genes can be categorized into seven primary clades, according to phylogenetic analysis. This analysis showed that all *HH3* genes are highly conserved and have evolved before the differentiation of monocots and dicots. The existence of *HH3* genes in each organism, with 17 *HH3* genes in *G. max* and just four genes in *V. vinifera*, revealed that *HH3* genes are evolutionarily conserved and have expanded widely in plants.

Multiple sequence alignment was utilized to construct sequence logos of conserved AARs for dicots (*A. thaliana* and *G. max*) and monocots (*O. sativa*). Furthermore, regardless of the N or C terminus, the sequence logos of all three identified species were largely conserved. Previous research has found that histone proteins are substantially conserved among studied plant species, despite the discovery of a number of variants depending on amino acid differences in their sequences. These variations could be as small as a few amino acids or as vast as a major percentage of a protein. The histone variation H3 has been linked to gene transcription in a favorable way. *HH3* enrichment was previously identified toward promoters and transcription termination sites in a genome-wide investigation (Stroud et al., 2012; Wollmann et al., 2012; Shu et al., 2014). In sequence logo analysis, the AARs such as M, A, R, Q, R, P, P, G, T, V, A, L, R, I, R, K, Y, Q, K, T, R, K, L, P, Q, A, V, A, and others were highly conserved.

All the *GmHH3* genes displayed a nearly identical pattern of *cis*-elements linked to soybean growth and development, as well as light and stress responses, in their promoter sequences. Several studies have shown that light has a significant impact on plant development. *Cis*-elements such as heat stress response elements (Díaz-Martín et al., 2005), abscisic acid (ABA) responsive elements (Narusaka et al., 2003), and dehydration-response elements (Song et al., 2005) have been identified in different organisms. More *cis*-elements such as ARE, CGTCA-motif, GARE-motif, and TGACG-motif were identified for different stress responses (Singh, Foley, and Oñate-Sánchez, 2002), Box 4, Box I, Box II, G-box, and GA-motif were identified for light responses. These elements are found in a number of *GmHH3* genes, with typical traits confirming their predicted activities in growth, development, hormonal, and abiotic stresses.

Except for a few, all *GmHH3* genes had very comparable gene structures and protein motif distributions, specifying that *GmHH3* genes were evolutionarily conserved. Introns were said to have an integral part in the evolution of many plant species based on the gene structure (Roy and Gilbert, 2006). It is well-known that there were more introns during the early growth phase, which experienced decline over time (Roy and Penny, 2007). These research studies claim that more advanced species’ genomes have fewer introns (Qanmber et al., 2019b). The creation of novel functions is aided by the presence of more or larger introns. Tandem duplications result in a rise in introns, which leads to the emergence of additional genes. As two *GmHH3* genes experience tandem duplication so that these genes have three or four introns. From 17 *GmHH3* genes, 12 genes have no introns. These findings were in line with those of past studies. The *GmHH3* gene family is relatively old, with introns lost over a period of time, showing the evolutionarily conserved activities of this gene family soybean growth and development, based on the lower number of introns.

Crop breeding merely on a morphological basis is comparatively ineffective (Ur Rehman et al., 2019), and effective selection using the SNP-based molecular markers will definitely put the breeding process on the fast-track (Rasheed et al., 2017). Genomic studies in soybean were dependent to some extent on comparative genomics approaches with other members of model organisms. At present, the release of soybean PAN-genome has revolutionized the approach and paved a smooth way for genomic studies in soybean. The absence of polymorphism in all the *GmHH3* genes except *GmHH3-3* is possibly due to allele fixation during evolution or domestication. The other probable reason might be the investigation of a smaller number of soybean accessions for the identification of polymorphic sites.

Since 1923, the soybean genetic gain is estimated to be ∼0.34 bu/ac (Rincker et al., 2014). This genetic gain has largely been achieved by breeding for grain yield. Advanced molecular breeding tools can certainly be helpful in the improvement of genetic gain. Fast forward genetic gain can be achieved by coupling the marker-assisted selection with a lower generation turnaround time period. In this study, *GmHH3-3-T* showed a significant association with thousand grain weight in both environments, suggesting that the use of this allele could be instrumental for the higher thousand grain weight selection. Gel-free KASP assays (high-throughput) can considerably fast-track soybean breeding programs. Application of the SNP-based functional markers will be more efficient for plant yield improvement and has been recommended by different researchers (Semagn et al., 2014; Rasheed et al., 2016). The gene identified here and the molecular marker developed here to identify the allelic variation might be helpful for marker-assisted breeding for higher thousand grain weight which can be utilized alone or in combination with the other reported functional markers.

## Conclusion

We identified 139 *HH3* genes in 12 different organisms. Phylogenetic analyses, gene structure, and motif analysis revealed the conserveness of *HH3* genes across the species. *Cis*-element analysis predicted the role of *HH3* genes in soybean growth and development and as well as in light response and various stress responses. Collinearity analysis indicated that the soybean *HH3* gene family had undergone WGD, segmental, and tandem duplication. Marker trait association analysis confirmed that *GmHH3-3-T* had superiority over *GmHH3-3-C* regarding thousand grain weight.

## Data Availability

The original contributions presented in the study are included in the article/Supplementary Materials; further inquiries can be directed to the corresponding authors.

## Supplementary Material

Supplementary Table 1 SNP information and KASP marker detail.

Supplementary Table 2 Recipe of GmHH3-3 KASP assay for 1x sample.

Supplementary Table 3 HH3 gene IDs of 12 species.

Supplementary Table 4 Biophysical properties of GmHH3 genes.

Supplementary Table 5 Collinearity/synteny and Ka/Ks values of GmHH3-3.

Supplementary Table 6 cis-elements in GmHH3-3.
